# Structure-Function Relationship of Transporters in the Glutamate–Glutamine Cycle of the Central Nervous System

**DOI:** 10.3390/ijms19041177

**Published:** 2018-04-12

**Authors:** Mariko Kato Hayashi

**Affiliations:** School of Medicine, International University of Health and Welfare, 4-3 Kozunomori, Narita, Chiba 286-8686, Japan; hayashim@iuhw.ac.jp; Tel.: +81-476-20-7784

**Keywords:** glutamate transporter, vesicular glutamate transporter, glutamine transporter, astrocyte, neuron

## Abstract

Many kinds of transporters contribute to glutamatergic excitatory synaptic transmission. Glutamate is loaded into synaptic vesicles by vesicular glutamate transporters to be released from presynaptic terminals. After synaptic vesicle release, glutamate is taken up by neurons or astrocytes to terminate the signal and to prepare for the next signal. Glutamate transporters on the plasma membrane are responsible for transporting glutamate from extracellular fluid to cytoplasm. Glutamate taken up by astrocyte is converted to glutamine by glutamine synthetase and transported back to neurons through glutamine transporters on the plasma membranes of the astrocytes and then on neurons. Glutamine is converted back to glutamate by glutaminase in the neuronal cytoplasm and then loaded into synaptic vesicles again. Here, the structures of glutamate transporters and glutamine transporters, their conformational changes, and how they use electrochemical gradients of various ions for substrate transport are summarized. Pharmacological regulations of these transporters are also discussed.

## 1. Introduction

Glutamate is the major excitatory neurotransmitter in our central nervous system. Vesicular glutamate transporters load glutamate into synaptic vesicles at presynaptic terminals. An action potential propagated to a presynaptic terminal triggers glutamate release into the synaptic clefts. The glutamate activates glutamate receptors on synaptic membranes. It is then removed by glutamate transporters to prepare for another signal. The glutamate clearance also prevents neuronal excitotoxicity caused by excess activation of glutamate receptors. Both neurons and astrocytes express glutamate transporters for glutamate uptake.

Glutamate is converted to glutamine by glutamine synthetase in astrocytes. Glutamine transporters on the plasma membranes of astrocytes and neurons mediate the transfer of glutamine from astrocytes to neurons. Glutamine is then converted back to glutamate in neurons by glutaminase, and then loaded into synaptic vesicles for another round of presynaptic release. This whole process is called the glutamate–glutamine cycle.

All transporters working in the glutamate–glutamine cycle are membrane proteins which have multiple transmembrane helices. These transporters are pseudo-symmetric and use an alternating access mechanism to transfer their substrates from one side of the membrane to the other. They utilize the electrochemical ion gradient between the membranes to transport against the concentration gradient. However, although all of the transporters in glutamate–glutamine cycle share these properties, their structures, conformational changes, and ion gradients used for their substrate transport are different. Their crystal structures reveal how they operate and how they are pharmacologically regulated.

## 2. Transporter Subtypes and Glutamate–Glutamine Cycle

**Figure 1:**
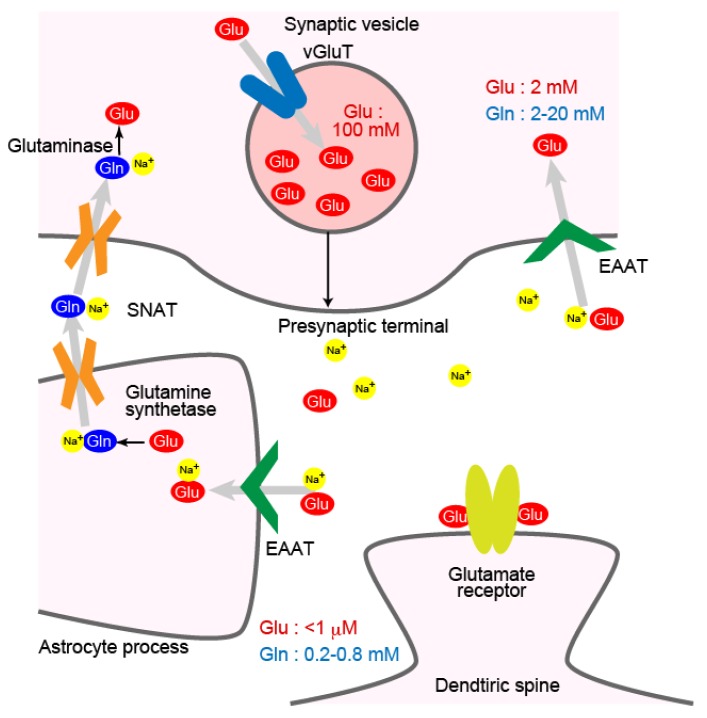
Localization of glutamate transporters and glutamine transporters around an excitatory synapse. Plasma membrane glutamate transporter EAATs are on the plasma membrane of presynaptic terminals and astrocyte processes. Vesicular glutamate transporter vGluTs are on synaptic vesicles. SNATs transports glutamine at the plasma membrane of neurons and astrocytes. Concentrations of glutamate and glutamine in each compartment are indicated.

Glutamate released from presynaptic terminals to the synaptic clefts is removed by glutamate transporters on the plasma membrane of neurons and astrocytes (Figure 1). Extracellular glutamate concentration around neurons at a quiescent state is kept below 1 μM, while its concentration at cytoplasm is much higher at around 2 mM [1]. The glutamate concentration in the cerebrospinal fluid is kept low by the blood–brain barrier, which restricts entry of the glutamate in blood plasma. Glutamate transporters use electrochemical gradient of sodium and potassium ions across the plasma membrane to transport glutamate against its concentration gradient [2].

Transporters that use electrochemical ion gradients across membranes for substrate transport are classified as secondary active transporters [3]. Among the secondary active transporters, glutamate transporters belong to the SLC1 (solute carrier 1) family of transporters. The human genome contains seven genes corresponding to the SLC1 family transporters. Of the seven, five are glutamate transporters called excitatory amino acid transporters 1–5 (EAAT1–EAAT5; SLC1A3, -2, -1, -6, and -7, respectively) [4]. In the case of rodents, GLAST (glutamate/aspartate-transporter) [5] corresponds to human EAAT1, and GLT1 (glutamate transporter 1) [6] corresponds to human EAAT2. For simplicity, this review will use EAAT1 and EAAT2 for GLAST and GLT1, respectively. The other two members of the SLC1 transporters are neutral amino acid transporters (ASCT1 and ASCT2; SLC1A4 and -5, respectively) named for transporting alanine, serine and cysteine as their substrates [7,8]. In addition, ASCT2 expressed in astrocytes mediates bidirectional sodium-glutamine antiport [9,10]. Besides these seven transporters, glutamate transporters do not share sequential or structural homology with other transporters [11,12].

The glutamate taken up by astrocytes through glutamate transporters is converted to glutamine by glutamine synthetase (Figure 1) [4]. Glutamine is released from astrocytes and then taken up by neurons. Unlike glutamate, glutamine does not affect neuronal activity when it is released from astrocytes. The glutamine is transported from astrocytes to neurons by transporters of SNAT (sodium-coupled neutral amino acid transporter; SLC38) family [13,14]. SNATs are members of the amino acid-polyamine-organocation (APC) transporter family. Many neurotransmitter transporters, such as the choline transporter, the dopamine transporter, the serotonin transporter or the noradrenaline transporter belong to this family. Glutamine transported back to neurons is converted to glutamate again by glutaminase [15].

At presynaptic terminals, vesicular glutamate transporters (vGluTs; SLC17A7, -6, and -8) load glutamate into synaptic vesicles (Figure 1) [16,17,18]. Their two subtypes, vGluT1 and vGluT2 are expressed in excitatory neurons in a complementary manner in the brain, defining two subsets of excitatory neurons [19]. Vesicular transporters for ATP, monoamine or acetylcholine are structurally similar to vGluTs. These vesicular transporters belong to the major facilitator superfamily (MFS) of proteins. Upon arrival of an action potential at a presynaptic terminal, the synaptic vesicles loaded with glutamate fuse to the presynaptic membrane to release the glutamate to the synaptic cleft.

## 3. Expression Profile of Plasma Membrane Glutamate Transporter EAATs

EAAT1 and EAAT2 are glutamate transporters mostly expressed in astrocytes. These two glutamate transporters are responsible for most of the glutamate clearance in the brain. EAAT2 is widely expressed in the cerebral cortex and the hippocampus. EAAT1 is strongly expressed in the cerebellar Bergmann glia cells that are of astrocyte lineage and surround Purkinje cells receiving excitatory inputs [20].

Neurons also express glutamate transporters. Five to ten percent of EAAT2 is expressed in neurons, and it is localized to neuronal axons and synaptic terminals [21]. In addition, EAAT3–5 are glutamate transporters mostly expressed in neurons. EAAT3 (also known as EAAC1, excitatory amino acid carrier 1) is located on the surface of neuronal cell bodies and dendrites [22,23]. EAAT3 is expressed at parvalbumin positive GABAergic interneurons [22,24], which receive excitatory inputs to their cell bodies. However, the expression level of EAAT3 only corresponds to 1% of EAAT2 in the brain. Parvalbumin positive neurons in the prefrontal cortex are required for executing tasks which need focus and attention [25]. EAAT4 is a neuronal glutamate transporter of the cerebellum. It is localized to perisynaptic surface of dendritic spines of cerebellar Purkinje cells. The surface of the Purkinje cells faces Bergman glia cell surface where the EAAT1 is localized [24,26,27]. EAAT5 is predominantly expressed in retinal photoreceptors and bipolar cells [28,29]. Both EAAT4 and EAAT5 show glutamate-gated chloride conductance in addition to sodium-dependent glutamate uptake [28,30]. This chloride conductance can counteract membrane depolarization caused by sodium influx during glutamate uptake.

Although EAAT2 on astrocytes plays a major role in glutamate uptake, contribution of neuronal EAAT2 is larger than what was expected from its mostly astrocytic expression. Cell-type-specific knockout studies show that astrocytic EAAT2 accounts for 80% of forebrain EAAT2 and glutamate uptake activity. EAAT2-knockout mice die early at ~6 weeks after birth due to seizures [31]. In contrast, astrocyte-specific EAAT2-knockout mice survive longer by 23 weeks on average, and do not show spontaneous seizures [32]. Neuron-specific EAAT2-knockout mice had normal life expectancies. The milder phenotype of astrocyte-specific EAAT2-knockout mice indicate that neuronal EAAT2 is sufficient to suppress seizures and to support survival.

## 4. Plasma Membrane Glutamate Transporter EAAT: Trimeric Transporters

### 4.1. Trimeric Structure of Glutamate Transporters

**Figure 2:**
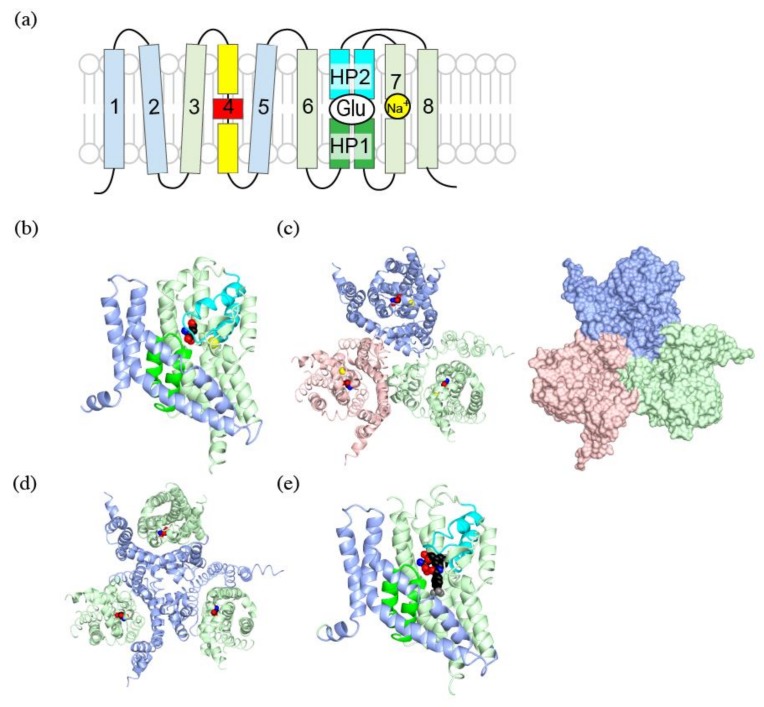
Crystal structures of a human EAAT1: (**a**) Topology diagram for SLC1 family transporters. The trimerization domain is in light blue; the transport domain is in light green; helical hairpin1 (HP1) is in green; HP2 is in cyan; and TM4 is in yellow and red; (**b**) A ribbon diagram of a human EAAT1 colored as in (**a**), with L-aspartate (spheres) and a sodium ion (yellow). PDBID:5LLU; (**c**) Ribbon (left) and surface (right) diagram of a human EAAT1 trimer viewed from extracellular face. Protomers of the trimer are colored in pink, light blue and light green; (**d**) Ribbon diagram of a human EAAT1 with the trimerization domain in light blue, and the transport domain in light green; (**e**) Ribbon diagram of a human EAAT1 with d,l-threo-b-benzyloxyaspartate (TBOA) (spheres) as shown in (**b**). Notice that the tip of HP2 (cyan) is open and does not cover TBOA. PDBID:5MJU. Pictures are generated using QtMG [40].

When glutamate transporters were first cloned, it was speculated that they have 8–10 transmembrane regions based on hydrophobicity of their amino acid sequence [6,33]. Crystal structures of SLC1 family transporters are extensively studied using GluTPh, a glutamate transporter of a thermophilic prokaryote *Pyrococcus horikoshii* [34]. Furthermore, crystal structures of human EAAT1 was solved to confirm the structural similarity of SLC1 family transporters across species [35]. Their crystal structures also clearly showed that SLC1 family transporters have eight transmembrane helices and a pair of helical hairpins, HP1 and HP2 (Figure 2a,b). The existence of helical hairpins apparently misled initial predictions of transmembrane regions from their primary structures. Both amino- (N-) and carboxy- (C-) termini of SLC1 family transporters are at cytoplasm and provide sites for interaction with cytoplasmic proteins [36,37,38,39]. 

The crystal structures reveal characteristic three-fold symmetric homotrimers of SLC1 family transporters (Figure 2c). The trimer forms a bowl facing extracellular environment, and its concavity penetrates halfway across the membrane bilayer. 

### 4.2. Trimerization Domain and Transport Domain Characterize Trimeric Transporters

Comparison of outward-facing and inward-facing structures of GluTPh reveals that the molecule can be divided into the trimerization domain and the transport domain which separately move during the transport [34,41]. Transmembrane (TM) helices 1, 2, 4 and 5 make up the trimerization domain (Figure 2a,b, light blue). Assembled as a trimer, they form a triangular propeller-like core structure (Figure 2d, light blue). Its structure, and most likely its position in the membrane bilayer, does not change during substrate transport [34,42]. The transport domain (Figure 2a,b light green) is composed of the pair of helical hairpins (Figure 2a, green and cyan) and their associating helices. The transport domain of each subunit (Figure 2d, light green) is inserted between the blades of the propeller of the trimerization domain (Figure 2d, light blue). A transport domain contacts exclusively with the trimerization domain of the same protomer and does not contact other protomers of the trimer (Figure 2c,d). The lack of interaction between the transporter domain and other protomers implies that each subunit of the trimer transports substrate independently.

### 4.3. Substrate Binding to the Transport Domain

The pair of helical hairpins of each transport domain forms a substrate-binding site. Each helical hairpin is composed of a helix-turn-helix motif. HP1 (Figure 2a,b, green) is inserted into the plasma membrane from the intracellular side, and HP2 (Figure 2a,b, cyan) is inserted into the extracellular side. The tips of helical hairpins meet at the bottom of the bowl and faces extracellular environment. The tips of the helical hairpins coordinate the transport substrate. 

A substrate bound to EAAT1 is secured under the tip of HP2 beneath the concave floor. The substrate is trapped by the transporter taking an outward-occluded conformation (Figure 2b, spheres) [35,43]. In the absence of a substrate, the HP2 is open exposing a pocket for the substrate binding, taking an outward-open conformation. The HP2 in the open conformation allows access of the substrate from extracellular environment [43].

Unlike transportable substrate complex in an outward-occluded conformation, EAAT1 and GluTPh with a non-transportable competitive blocker TBOA take an outward-open conformation [35,43] (Figure 2e). TBOA inhibits the transport by keeping HP2 in the open conformation (Figure 2e. cyan), preventing further conformational changes necessary for the substrate transport. 

## 5. Structural Changes During the Substrate Transport

### 5.1. Transporter Domain Movements During Substrate Transport

**Figure 3:**
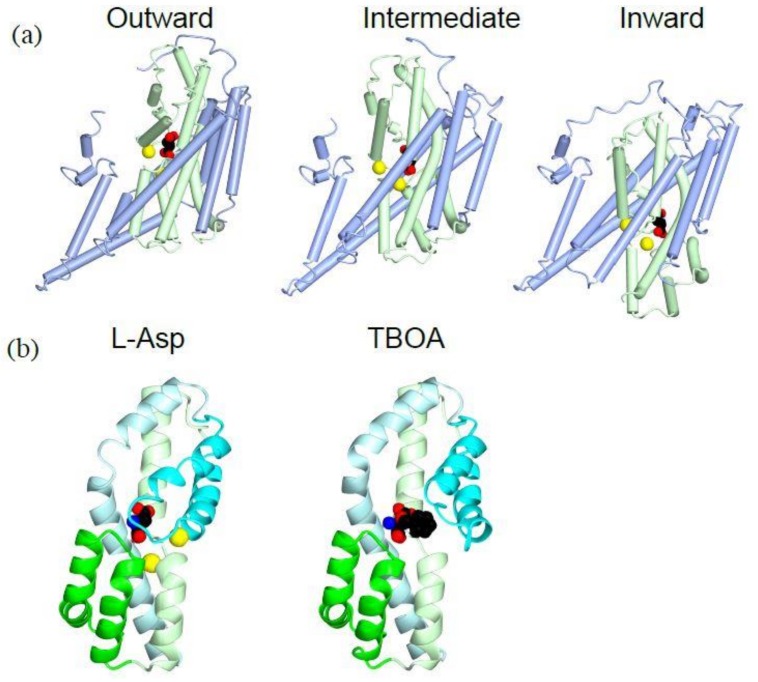
Crystal structures of GluTPh showing transport mechanism of a glutamate transporter: (**a**) Tube and worm diagrams showing the three stages of GluTPh during transport. Left, outward-occluded, PDBID:2NWX; middle, intermediate, PDBID 3V8G; right, inward-occluded, PDBID:3KBC. The trimerization domain is in light blue, and the transport domain is in light green with l-aspartate (spheres) and a sodium ion (yellow). The orientations of the trimerization domain of each structure are aligned. (**b**) Ribbon diagram of a transport domain core structure with HP1 (green), HP2 (cyan), TM7 (light green) and TM8 (light cyan). Left, l-aspartate (spheres) and sodium ions (yellow), PDBID:2NWX; right, TBOA (spheres), PDBID:2NWW.

The inward-facing structures of GluTPh were solved using a double cysteine mutant. These cysteine residues crosslink to stabilize the inward-facing conformation [41]. During the transition from the outward-facing conformation to the inward-facing conformation, the transport domain moves 18 Å towards the cytoplasm (Figure 3a, light green). Both the transport domain and the trimerization domain moves as separate rigid bodies (Figure 3a). Because of the movement, the substrate-binding site is located near the cytoplasmic face of the membrane bilayer in the inward-facing conformation (Figure 3a, right, sphere). The substrate-binding site is occluded from intracellular solution by the tips of HP1 and HP2, taking an inward-occluded conformation. It is likely that HP1 moves to open the gate and allows dissociation of the substrate to cytoplasm. An intermediate structure during the transport indicate that each protomer of a trimer can take different conformations representing different stages of substrate transport [44]. This is more evidence that the transport is not coordinated among the subunits of the trimer.

### 5.2. Coordination of Sodium Ions for Co-Transport

As secondary active transporters, glutamate transporters use electrochemical gradient of sodium, potassium and proton across the plasma membrane for substrate transport. EAAT3 co-transports three sodium ions and one proton with each glutamate into the cell, and transports one potassium ion out from the cell [2]. Using the ion gradients across the plasma membrane, glutamate is imported to the cytoplasm against its concentration gradient. 

There are two sodium ions near the substrate-binding site of GluTPh (Figure 3b, yellow). HP2 (cyan), TM7 (light green) and TM8 (light cyan) of the transport domain coordinate the two sodium ions. TM7 (light green) has an unwound helical part to coordinate one of the sodium ions. The structure shows that the two sodium ions bind to the transporter cooperatively with the substrate. The cooperativity is consistent with sodium-dependent substrate binding to the transporter [34]. The sodium binding stabilizes the HP2 forming an extracellular gate in a closed conformation (Figure 3b, left, cyan). Remarkably, when a competitive blocker TBOA is bound to the transporter, the second sodium ion does not bind and the extracellular gate HP2 is kept open (Figure 3b, right, cyan). 

The cooperative binding of sodium ions and the substrate shows how sodium import is coupled to glutamate uptake. Sodium concentration is high in the extracellular fluid and low in the cytoplasm. This difference is responsible for the different affinity of glutamate at each side of the plasma membrane. Glutamate transport against the concentration gradient between the plasma membrane is possible because of the effective concentration of sodium-glutamate being lower in the cytoplasm than in the extracellular environment.

### 5.3. Interaction with Lipids Affect the Transporter Activity

**Figure 4:**
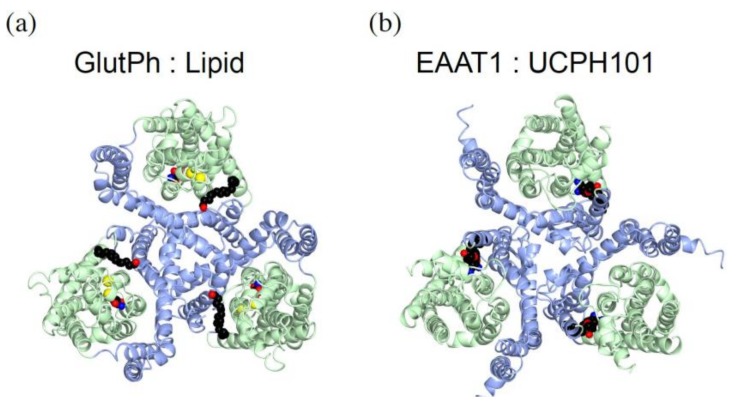
Glutamate transporter inhibitors bound at the domain interface. The trimerization domain is in light blue, and the transport domain is in light green with L-aspartate (spheres) and a sodium ion (yellow). (**a**) GluTPh with a lipid or detergent molecule (spheres, red/black) between domain interfaces. PDBID:2NWX; (**b**) EAAT1 with a non-competitive inhibitor UCPH101 (spheres). PDBID:5MJU.

One of the GluTPh crystal structures revealed non-protein electron densities at the interface between the trimerization domain and the transport domain [34] (Figure 4a, spheres). They are likely to be detergent or lipid molecules. Some lipids affect glutamate transporter activity [45]. Lipids are located at the hydrophobic crevices at the interfaces of the trimerization domain and the transport domain. This indicates that the lipids affect the movement of the transport domain during the substrate transport.

UCPH101, a non-competitive blocker of EAAT1, also binds near the domain interface. The location is close to where lipids bind to in GluTPh trimer (Figure 4b, spheres). UCPH101 interacts with both the trimerization domain and the transport domain, but binding sites are more than 15 Å away from the substrate- and sodium-binding sites. UCPH101 inhibits substrate transport by trapping the transporter in an outward-facing conformation and by inhibiting the transport domain translocation. 

### 5.4. Transport Kinetics of Glutamate Transporters

Single-molecule fluorescence resonance energy transfer imaging shows that GluTPh preferentially takes outward-facing conformations while sometimes adopting less favorable inward-facing orientation, indicating the shuttle movement of the transport domain [42]. Observation of intermediate states indicates that neighboring subunits can take inward- and outward-facing orientations independently. It seems that the transport domain movements across the plasma membrane occur spontaneously. Outward-facing orientations are preferred in the presence of substrate and sodium ions. In addition, substrate binding delays the transitions between outward- and inward-facing conformations by more than ten times. This transition may involve dislodging of the transport domain from the trimerization domain before the movement [44].

### 5.5. TM4 of the Trimerization Domain Has an Insertion of an Extracellular Loop

**Figure 5:**
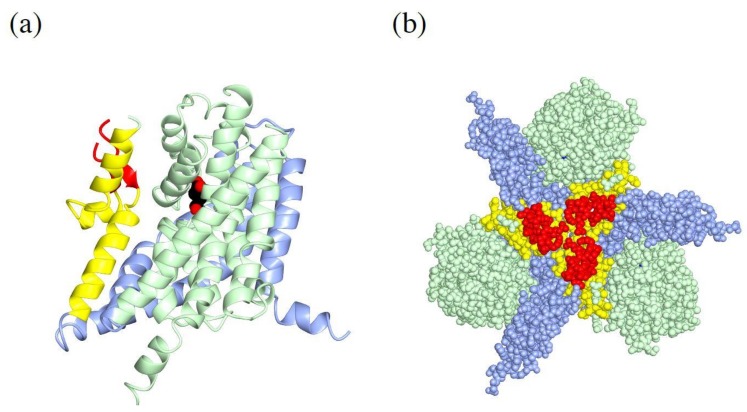
Structure of EAAT1 TM4: (**a**) Ribbon diagram of an EAAT1 monomer. The trimerization domain is in light blue, TM4 is in yellow, and the loop of TM4 unique for metazoans is in red. The transport domain is in light green with l-aspartate (spheres). PDBID:5LLU. (**b**) Sphere diagram of EAAT1 trimer, colored as in (**a**).

TM4 of the trimerization domain takes a complex helix-turn-helix-turn-helix structure (Figure 5a, yellow). The TM4 is located at subunit–subunit interface (Figure 5b, yellow). The second turn of the TM4 has an insertion loop for SLC1 family transporters of metazoans, but not for unicellular organisms. This loop is located near the center of the concavity of a trimer (Figure 5b, red). Only a part of the loop is visible in the crystal structure of EAAT1. All human SLC1 family transporters have multiple highly glycosylated *N*-glycosylation sites in this loop, but the glycosylation is not required for glutamate transport [46]. 

Glutamate transporters localize to astrocyte processes [47,48]. The transmembrane domain fragments without cytoplasmic tails are sufficient for localization to cellular process tips. The extracellular loop of TM4, but not its glycosylation, is required for the cellular process localization [49]. The crystal structure indicates that the loop extending toward extracellular environments interact with some extracellular matrix molecules to regulate the localization of glutamate transporters.

## 6. Structure of Plasma Membrane Glutamine Transporters: APC Family Transporters

After glutamate is taken up by astrocytes, it is converted to glutamine by cytosolic glutamine synthetase [50,51]. SNAT3 and SNAT5 on astrocyte plasma membrane allow glutamine efflux from cytosol [52]. The SNATs work as sodium symporters and proton antiporters for glutamine export. The sodium ions imported to cytosol with glutamate through EAATs can be exported with glutamine through SNATs. SNAT3 forms a complex with EAAT1, indicating their physical and functional coupling [53]. On the neuronal plasma membrane, SNAT1, SNAT2 and SNAT7 mediate glutamine uptake as sodium symporters [54].

SNATs belong to APC superfamily of transporters, the second largest superfamily of secondary active transporters [14,55]. Many neurotransmitter transporters on the plasma membrane, but not glutamate transporters, belong to this family. Among these are the serotonin transporter, the dopamine transporter, the norepinephrine transporter, and the GABA (γ-aminobutyric acid) transporters. These neurotransmitter transporters are sodium symporters.

**Figure 6:**
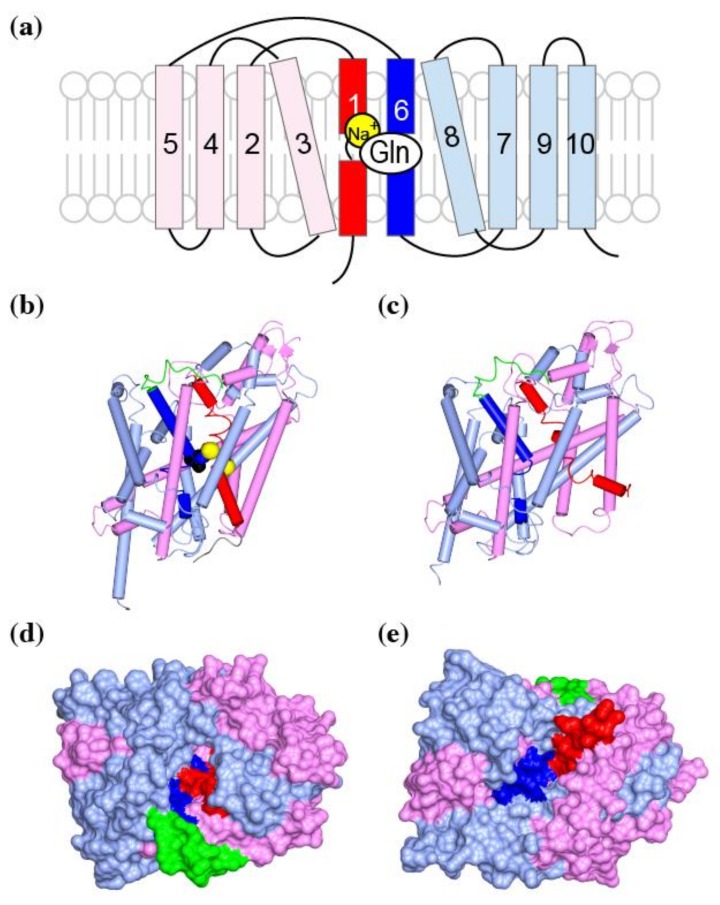
Structure of LeuT, a bacterial homolog of the glutamine transporter SNATs: (**a**) Topology diagram of APC family transporter domain. TM1 is in red, TM2–5 are in pink, TM6 is in blue, and TM7–10 are in light blue; (**b**) A worm and tube diagram of LeuT colored as in (**a**), in a substrate leucine (spheres) bound, outward-occluded conformation. The fourth extracellular loop is in green, and the sodium ions are yellow spheres. PDBID:2A65; (**c**) A surface representation of (**b**) from the extracellular side. The pocket for substrate binding is occluded by TM1 (red) and TM6 (blue); (**c**) LeuT in an inward-open conformation, colored as in (**a**). PDBID:3TT3; (**d**,**e**) Surface representations of (**b**,**c**) from the extracellular or intracellular side of the molecule, respectively.

Secondary active transporters transport substrate by alternating access mechanism. These transporters alternatively switch from the outward-open form to the inward-open form, and transport their substrate across the membrane. One example is the SLC1 family transporters which transports their substrates by using shuttle movements of the transport domain. The Crystal structures of the bacterial APC superfamily transporter LeuT reveals another alternating access transport mechanism. The APC superfamily transporters consist of ten to twelve transmembrane helices. Among these helices, TM1–5 and TM6–10 form two subdomains with structural similarity. The two subdomains are arranged around horizontal pseudo-2-fold-symmetry axis (Figure 6, pink and red for TM1–5 and light blue and blue for TM6–10) [56]. The substrate (spheres) and sodium ions (yellow) are coordinated by two partially unwound transmembrane helices of TM1 (red) and TM6 (blue). In its outward-occluded conformation, the fourth extracellular loop (green) covers the substrate to avoid escape to extracellular fluid (Figure 6a, green). The loop must move for the substrate to enter the substrate-binding pocket. During transition from the outward-facing to the inward-facing conformation, the TM1 and TM6 coordinating the substrate makes a hinge-bending movement (Figure 6b,c, red and blue). These changes close the extracellular gate and opens the intracellular gate to allow dissociation of the substrate to the cytoplasm. In the inward-facing conformation, the structure of residues coordinating two sodium ions are disordered to release the substrate and ions to the cytoplasm [57].

## 7. Structure of Vesicular Glutamate Transporters: MFS Transporters

Vesicular glutamate transporters are MFS transporters, which represent the largest secondary active transporter family [58,59,60]. Vesicular transporters for monoamine, acetylcholine or ATP are also MFS transporters. In contrast, GABA or glycine are loaded to synaptic vesicles by APC family transporters [60,61,62]. Vacuolar-type H^+^-ATPase pumps protons into synaptic vesicles to generate electrochemical gradient of proton. The chemical gradients of proton and chloride, and membrane potential are used to load glutamate into synaptic vesicles to the concentration as high as 100 mM [1,63,64]. Unfortunately, no crystal structures of vesicular neurotransmitter transporters are known to date. Among human MFS transporters, crystal structures are available for human GLUTs (glucose transporters; SLC2A), in both outward [65] and inward-facing [66] conformations showing that MFS transporters also transport substrates using an alternating access mechanism. 

**Figure 7:**
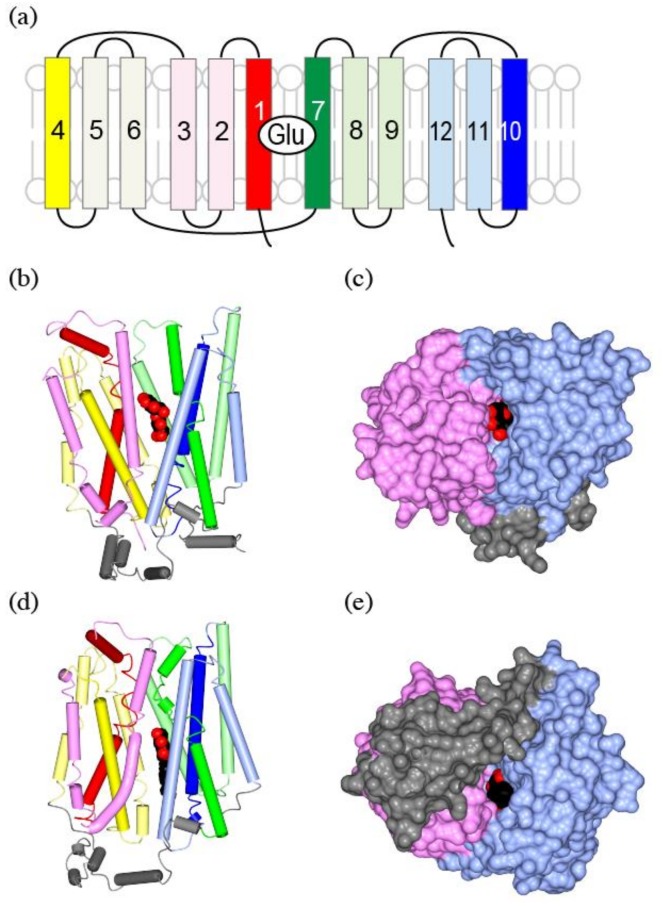
The crystal structure of GLUT, a human glucose transporter as a homolog of vGluT: (**a**) Topological structure of GLUT. The N-terminal domain is in warm colors (red to yellow), while the C-terminal domain is in cool colors (green to blue). TM1 is in red, TM2–3 are in pink, TM4 is in yellow, TM5–6 are in lemon, TM7 is in green, TM8–9 are in light green, TM10 is in blue, and TM11–12 are in light blue; (**b**) A worm and tube diagram of GLUT3 with substrate glucose (spheres) bound, outward-occluded conformation. The substrate leucine as spheres, and sodium ions as yellow spheres. PDBID:2A65; (**c**) A Surface representation of (**b**) from the extracellular side of the molecule. The N-terminal domain is in pink, and the C-terminal domain is in light blue; (**d**) LeuT in an inward-open form, colored as in (**a**). The bound ligand is 1-S-octyl-b-D-thioglucoside (spheres), a detergent with a sugar group used in the sample preparation. PDBID: 3TT3; (**e**) Surface representation of (**d**) from intracellular side of the molecule. Intracellular helices in gray are unique for glucose transporters, and are missing in vesicular neurotransmitter transporters.

MFS transporters have 12 transmembrane helices. The molecule can be divided into the N-terminal domain and the C-terminal domain, each with six transmembrane helices. These two domains are structurally similar [67,68] and they are related by a vertical two-fold pseudo-symmetry axis (Figure 7a,b, warm colors at left and cool colors at right). Each half can be further divided into tandem repeats of three-helix bundles (Figure 7a,b, red–pink, yellow–lemon, green–light green, and blue–light blue). In the case of GLUT, the substrate mostly interacts with the C-terminal domain (Figure 7b, cool colors), and the two discontinuous helices of TM7 (Figure 7a,b, green) and TM10 (Figure 7a,b, blue) provide major residues for substrate coordination.

During the switch between the outward-facing (Figure 7b,c) and inward-facing conformations (Figure 7d,e), the N-terminal domain (Figure 7, warm colors) and the C-terminal domain (Figure 7, cool colors) are rotated by 15 degrees relative to each other (Figure 7b,d). The N-terminal domain rotates as a rigid body, while the discontinuous helices TM7/10 of the C-terminal domain coordinating the substrate cause some local rearrangements.

Translocation of protons during transport involves protonation and deprotonation of acidic or basic residues. Structure of proton-dependent bacterial transporters and their mutagenesis studies indicate that TM1, -4, -7, and -10, the first helices of each three-helix bundle (Figure 7a,b,d, red, yellow, green, and blue), are responsible for the proton dependent transport [58].

## 8. Regulation of Synaptic Transmission by Glutamate Transporters

### 8.1. Glutamate Transporters Shape Synaptic Transmission

Unlike acetylcholine or monoamine neurotransmitters, which are enzymatically degraded, glutamate is not enzymatically degraded in the synaptic clefts. Therefore, diffusion from the synaptic clefts and clearance through glutamate transporters determine the timecourse of glutamatergic responses.

Glutamate is released into the narrow synaptic clefts, where astrocyte processes do not penetrate. At the synaptic clefts, diffusion and dilution of glutamate determines the concentration around synaptic glutamate receptors. Therefore, glutamate transporters do not affect the response mediated by synaptic AMPA (α-amino-3-hydroxy-5-methyl-4-isoxazolepropionic acid)-type glutamate receptors. AMPA receptors have lower affinity for glutamate than NMDA (*N*-methyl-d-aspartate)-type glutamate receptors and only respond to glutamate released at the synapses. In contrast, extrasynaptic NMDA receptors or metabotropic glutamate receptors can respond to glutamate spilled-over from the synapses and their responses are affected by activity and localization of glutamate transporters [69,70]. 

Activation of extrasynaptic NMDA receptors, caused by the glutamate that spilled over from synapses, affects glutamate excitotoxicity. The extrasynaptic concentration of glutamate is affected by glutamate uptake through glutamate transporters. Therefore, either the loss or upregulation of EAAT2 affects synaptic plasticity mediated by NMDA receptors or metabotropic glutamate receptors [71,72].

### 8.2. Glutamatergic Neurotransmission Affect Glutamate Transporters

Not only glutamate transporters affect glutamatergic synaptic transmission, but glutamatergic synaptic transmission also influences glutamate transporters. Single particle tracking of EAAT2 showed that its diffusion on the plasma membrane was restricted around glutamatergic synapses. Adding glutamate increased surface diffusion of EAAT2, while the application of its inhibitor, TBOA, reduced its mobility [73]. Photo-uncaging of glutamate around the synapse, mimicking synaptic glutamate release, increase the mobility of EAAT2 around the synapse [73]. 

Glutamate uptake is regulated by neuronal activity. After a burst of neuronal activity, glutamate uptake is reduced for a brief period of 50 ms around activated synapses. This reduction depends on presynaptic neuronal activity, but it does not depend on the amount of glutamate released. This reduction in glutamate uptake prolongs the duration of NMDA receptor currents [74]. 

### 8.3. Glutamate Transporters and Astrocyte Morphology

Fine tube- or sheet-like astrocyte protrusions called perisynaptic astrocytic processes (PAPs) reach out to excitatory synapses. However, not all glutamatergic synapses are approached by astrocyte processes. Serial electron microscopy showed that only about half of the glutamatergic synapses in the CA1 stratum are approached by astrocyte processes [75]. Synaptic activity, inducing long-term potentiation, increases PAP extension and synapse coverage, and stabilizes dendritic spines [76]. 

A gap junction protein connexin 30 is a regulator of cell adhesion. It is required for inserting PAPs into synaptic clefts and facilitating the glutamate clearance by astrocytes. Loss of connexin 30 impairs excitatory synaptic transmission and synaptic plasticity [77]. The role connexin 30 shows the importance of glutamate clearance at PAPs in regulating excitatory signals.

EAAT2, EAAT1 and glutamine synthetase are locally synthesized at PAPs [78]. The glutamate transporters that are locally synthesized at PAPs are inserted into the PAP membrane for glutamate uptake. The local synthesis of glutamine synthase supports the local glutamate–glutamine cycle, which is the predominant pathway for excitatory neurotransmitter recycling [79]. The product glutamine is then transported back to neurons through SNATs, which form a complex with glutamate transporters. This way, glutamate uptake is coupled to glutamine synthesis and export.

### 8.4. Transcriptional Regulation of Glutamate Transporters

Neurons release glutamate as a neurotransmitter and affect the expression of glutamate transporters in astrocytes. Astrocytes in monoculture express EAAT1, but EAAT2 expression is induced by neurons [80]. Both neuron-conditioned medium [81] and contact with neurons [82] can upregulate EAAT2 expression. The EAAT2 induction by neurons requires nuclear factor-κB (NF-κB), which binds to EAAT2 promoter. In addition, κB-motif binding phosphoprotein (KBBP), also known as nuclear ribonucleoprotein K (hnRNP K) binds to the EAAT2 promoter. Neuron-stimulated KBBP transcription is required for EAAT2 transcriptional activation. Conversely, neurodegeneration reduces KBBP expression in astrocytes, which results in reduced transporter expression [82]. 

Astrocyte stellation and glutamate transporter expression are also controlled by cAMP. Treatment of astrocytes with its non-hydrolysable analog dibutyryl-cAMP increases expression of EAAT1 and EAAT2, and facilitates formation of multiple branches [81].

In addition to using drugs that directly activate glutamate transporters, increasing glutamate transporter expression is a possible pharmacological approach to counteract glutamate excitotoxicity. β-lactam antibiotics are inhibitors of bacterial cell wall biosynthesis. Many β-lactam antibiotics enhance transcription of EAAT2. These β-lactams are neuroprotective in an ischemic injury model of oxygen-glucose deprivation, and delay loss of motor neurons in genetic mouse models mice of amyotrophic lateral sclerosis (ALS) [83]. It is likely that these β-lactam antibiotics enhance EAAT transcription by promoting nuclear translocation of p65 subunit of NF-κB [84]. 

ALS is a neurodegenerative disease associated with impairment of EAAT2 in some patients [85]. Preventing or retarding neurodegeneration in ALS patients using neuroprotective drugs is one possible strategy for its treatment. Riluzole, which activates glutamate transporters, is approved for ALS treatment in the United States [86,87]. However, ceftriaxone, the most promising β-lactam for neuroprotection, was effective for rodents, but clinical trials for ALS treatment were unsuccessful [88]. 

The female sex hormone estrogen also has neuroprotective effects [89]. Estrogen receptor activation upregulates EAAT2 and EAAT1 expression. The action is partly mediated by transforming growth factor-α and NF-κB [90]. Estrogen treatment of human astrocytes derived from cortex of Alzheimer's disease patients successfully restored glutamate transporter expression [91]. However, adverse effects of long-term estrogen usage are a potential problem for clinical application. 

For the survival of neurons, it is better if the activity of glutamate transporters is enhanced when neurons are challenged by excitotoxicity. However, glutamate transporters transport glutamate bidirectionally. Glutamate transporters of both neurons and astrocytes became the route for the leak of cytoplasmic glutamate into extracellular space as a source for glutamate under severe ischemia [92,93]. Therefore, we must consider the effect of glutamate efflux through glutamate transporters when we try to protect neurons by regulating glutamate transporters.

### 8.5. Pathophysiology of Glutamate Transporters

Neuronal death due to glutamate excitotoxicity or an imbalance of excitatory and inhibitory neuronal activity underlies many neurodegenerative or psychiatric diseases. It has been proposed that abnormal excitatory/inhibitory input ratio underlies neuropsychiatric disorders, such as autism spectrum disorders [94], obsessive-compulsive disorder [95], and schizophrenia [96]. A decrease in EAAT2 has been reported in the motor cortex of amyotrophic lateral sclerosis patients [97]. Single nucleotide polymorphism has been reported for EAAT2 genes in schizophrenia [98], bipolar disorder [99], and stroke [100] patients. 

Behavioral studies of glutamate transporter knockout mice support the hypothesis that dysfunction of glutamate transporters underlies these psychiatric diseases. For example, EAAT1-knockout mice show schizophrenia-like phenotypes [101]. In addition, astrocyte-specific EAAT2 inducible knockout mice exhibit pathological repetitive behaviors, which is one of the symptoms of obsessive-compulsive disorders or autism spectrum disorders. The repetitive behaviors were effectively treated with NMDA receptor antagonist memantine [102].

## 9. Conclusions

Glutamate transporters and glutamine transporters working in the glutamate–glutamine cycle are secondary active transporters and they use electrochemical gradient of ions between the membranes for transporting the substrate, mostly, against its concentration gradient. Furthermore, they transport the substrate across the membrane by alternatively switching from an outward-open form to an inward-open form. However, structures of the glutamate transporters and their conformational changes responsible for substrate transport differ among them despite the commonality or similarity of their substrates.

## Abbreviations

EAAT	excitatory amino acid transporter
GLAST	glutamate/aspartate-transporter
GLT1	glutamate transporter 1
ASCT	neutral amino acid transporter
SLC1	solute carrier 1
MFS	major facilitator superfamily
APC	amino acid-Polyamine-organoCation
TBOA	d,l-threo-b-benzyloxyaspartate
AMPA	α-amino-3-hydroxy-5-methyl-4-isoxazolepropionic acid
NMDA	*N*-methyl-d-aspartate
SNAT	sodium-coupled neutral amino acid transporter
vGluT	vesicular glutamate transporter
NF-κB	nuclear factor-κB
KBBP	κB-motif binding phosphoprotein
